# Phase-Amplitude Coupling Is Elevated in Deep Sleep and in the Onset Zone of Focal Epileptic Seizures

**DOI:** 10.3389/fnhum.2016.00387

**Published:** 2016-08-03

**Authors:** Mina Amiri, Birgit Frauscher, Jean Gotman

**Affiliations:** ^1^Montreal Neurological Institute, McGill University, MontrealQC, Canada; ^2^Department of Medicine and Center for Neuroscience Studies, Queen’s University, KingstonON, Canada

**Keywords:** epilepsy, cross-frequency coupling, phase- amplitude coupling, seizure onset zone, sleep, interictal EEG

## Abstract

The interactions between different EEG frequency bands have been widely investigated in normal and pathologic brain activity. Phase-amplitude coupling (PAC) is one of the important forms of this interaction where the amplitude of higher frequency oscillations is modulated by the phase of lower frequency activity. Here, we studied the dynamic variations of PAC of high (gamma and ripple) and low (delta, theta, alpha, and beta) frequency bands in patients with focal epilepsy in different sleep stages during the interictal period, in an attempt to see if coupling is different in more or less epileptogenic regions. Sharp activities were excluded to avoid their effect on the PAC. The results revealed that the coupling intensity was generally the highest in stage N3 of sleep and the lowest in rapid eye movement sleep. We also compared the coupling strength in different regions [seizure onset zone (SOZ), exclusively irritative zone, and normal zone]. PAC between high and low frequency rhythms was found to be significantly stronger in the SOZ compared to normal regions. Also, the coupling was generally more elevated in spiking channels outside the SOZ than in normal regions. We also examined how the power in the delta band correlates to the PAC, and found a mild but statistically significant correlation between slower background activity in epileptic channels and the elevated coupling in these channels. The results suggest that an elevated PAC may reflect some fundamental abnormality, even after exclusion of sharp activities and even in the interictal period. PAC may therefore contribute to understanding the underlying dynamics of epileptogenic brain regions.

## Introduction

Rhythmic changes in cortical excitability lead to neuronal oscillations with different spatial scales and therefore different cell population sizes (Fries, 2005). Low-frequency rhythms are involved in interacting activity over large spatial regions and in long temporal windows, while high-frequency rhythms are involved in local neural interactions and short temporal windows and are unlikely to contribute in distal structure integrations (von Stein and Sarnthein, 2000). The interactions between anatomical structures and oscillatory patterns allow the brain to work simultaneously at multiple temporal and spatial scales (Buzsaki and Draguhn, 2004). The different neuronal oscillations are not independent and isolated; they can interact with each other, and can modulate the oscillations in other frequency bands (Jensen and Colgin, 2007). This interaction between different frequency bands is called cross frequency coupling (CFC), which can include phase synchronization, amplitude co-modulation and phase-amplitude coupling (PAC; Pittman-Polletta et al., 2014).

Phase-amplitude coupling is believed to reflect neural coding and information transfer within the local microscale and macroscale neural ensembles of the brain (von Stein and Sarnthein, 2000; Vanhatalo et al., 2004; Jensen and Colgin, 2007; Axmacher et al., 2010; Canolty and Knight, 2010). Low frequency oscillations are assumed to regulate information between areas by modulating excitability of local ensembles (von Stein and Sarnthein, 2000; Fries, 2005), and their phase affects rhythmic high frequency activity, and the spiking rates of individual neurons (Canolty and Knight, 2010). PAC therefore facilitates effective interactions between neurons with similar phase preferences, and strengthens the synchronization of high frequency bands during specific phases of the slower rhythm (Allen et al., 2011).

There are several hypotheses underlying PAC. For instance, PAC could develop from pulses of inhibition paced at the slower frequency and targeted at the generators of the faster oscillation (Wulff et al., 2009; Tort et al., 2013). Moreover, the modulations of brain waves control baseline excitability and for each band of oscillation, there are both facilitating and inhibitory phases, during which stimulus responsiveness is enhanced or weakened (Lakatos et al., 2005). Perisomatic basket cells fire theta rhythmic trains of action potentials at gamma frequency, and are also thought to be a generator of PAC (Buzsaki and Wang, 2012).

The coupling between slow waves and high frequency activity (above 80 Hz) has been the subject of many studies considering that there is some evidence that the phase of slow waves can influence high frequency oscillations (HFOs) generation: the role of inhibitory control over interneurons in the coupling of hippocampal rhythms across different frequency bands has been investigated and it is shown that synaptic inhibition shapes both low and HFOs and could also control CFC (Bragin et al., 1995; Bartos et al., 2002; Wulff et al., 2009). Furthermore, low frequency phase entrainment shifts the relative timing of action potentials and synaptic activity dynamic which are phase-locked to ripple cycles (Canolty and Knight, 2010). Developing computational models, it has also been shown that one of the factors that could impact the HFO generation is alteration of inhibitory drive onto pyramidal neurons from interneurons (Wendling et al., 2012).

These findings motivated PAC research not only in healthy people but also in epileptic patients. In one of the studies on epileptic patients, Nariai et al. (2011) have shown that ictal and interictal HFOs in the seizure onset site are coupled with different frequency bands. In another study, Ibrahim et al. (2014) have investigated PAC in seizure onset zone (SOZ), early propagation zone and non-epileptic cortex during seizure and non-seizure intervals, and have shown that HFOs are modulated by the phase of alpha band in SOZ. PAC has also been used in seizure prediction studies. Alvarado et al. (2014) have illustrated that the modulation of high-gamma activities by slow waves identify preictal changes in a few patients. Furthermore, in a recent study it was observed that cross-channel PAC (delta-HFO) increases during seizures and can localize the necessary resection area for a majority of studied patients (Guirgis et al., 2015). Maris et al. (2011) investigated whether PAC plays a role in the working memory operations in epileptic patients. They concluded that working memory operations affect the PAC strengths in a heterogeneous way. A study of our group on slow waves and HFOs revealed that spikes and HFOs were more frequent during high-amplitude slow waves, and HFOs in channels without epileptic activity peak at a different phase of the slow wave cycle from those in channels with epileptic activity (Frauscher et al., 2015).

The modulation of epileptic activity by different sleep stages has been the focus of several studies. For instance, comparing to the waking state, interictal epileptiform discharges (IEDs) showed an increase in light and in deep sleep but not significantly in rapid eye movement (REM) sleep (Gloor et al., 1958). During REM sleep, IEDs and HFOs were found to predominate during sections without REMs compared to sections with REMs (Frauscher et al., 2016). It is also shown that rates of HFOs are highest in non-REM sleep, and HFOs, particularly fast ripples, provide a more reliable indicator of the SOZ in this stage (Bagshaw et al., 2009). The coupling between slow wave sleep and higher frequency activity has also been investigated widely for both human and animal models, showing that during sleep, the emergence of HFO during slow oscillations facilitates the communication among specific brain regions (Nagasawa et al., 2012; Valderrama et al., 2012; Valencia et al., 2013). However, the role of vigilance states in CFC has been neglected in most studies. The strength of CFC may vary during different states of vigilance and sleep, and therefore, it should be carefully studied to avoid misinterpretation of the physiological changes (caused by changing of sleep stages) for epileptic coupling alteration in preictal and ictal periods.

In this study, we will analyze the local PAC (i.e., the low and high frequency activity is measured in the same brain location) inside and outside of the SOZ in interictal periods in patients with focal epilepsy. We think that the epileptogenic region may have abnormal local PAC because of its disorganized neurons. This may also result in abnormal long-distance PAC between different brain regions (but this is not the purpose of this study).We will also investigate the variation of PAC between high (30–260 Hz) and lower frequency bands (0.3–30 Hz) during different sleep stages (N1, N2, N3, and REM).

## Materials and Methods

### Data

We studied the data from consecutive epileptic patients that underwent an intracerebral depth electrode (SEEG) study for presurgical evaluation at the Montreal Neurological Institute and Hospital between October 2011 and May 2015, with additional subcutaneous scalp electrodes for sleep scoring (sampled at 2000 Hz). This study was approved by the Review Ethics Board at the Montreal Neurological Institute and Hospital, and all patients signed an ethical board approved written informed consent. The inclusion criteria were:

1. Having pharmaco-resistant focal epilepsy with at least one continuous whole-night polysomnography with preferably additional electrooculography and chin electromyography (for more details see Frauscher et al., 2016), obtained at least 72 h after electrode implantation.
2. Having data recorded during sleep away from generalized seizures for at least 12 h, and away from partial complex seizures for at least 6 h.
3. EEG sampling of SOZ, exclusively irritative zone (EIZ, spiking channels outside SOZ) and presumably normal zone (NoZ, channels without any epileptic activity or any other anomalies, and out of SOZ) during the usual 2 week intracerebral recording).
4. No epileptic activity or non-epileptic anomalies interfering with sleep scoring in the scalp EEG.

From 46 studied patients, 25 fulfilled the inclusion criteria. The 21 exclusions were due to presence of seizures within the required seizure-free period (8), lack of normal channels (7), lack of identification of the seizure generator (3), interference of IEDs with sleep scoring (2), and lack of a 72 h interval after electrode implantation (1). Demographic and clinical data of investigated patients are provided in Supplementary Table S1. An average of eight depth electrodes (range: 4–13) was implanted using an image-guided system. In 17 patients, electrodes manufactured on site were implanted (nine contacts, 0.5–1 mm in length and 5 mm apart); and in eight patients, commercially available electrodes were used (DIXI Medical, France: 10–18 contacts, 2 mm in length and 1.5 mm apart). We analyzed all the available channels, and did not exclude any except for channels with major non-epileptic abnormalities and artifacts, and channels outside the brain (superficial channels located in CSF or bone that do not record the signal from the brain tissue). Sleep scoring was done manually in 30-s epochs according to the criteria of the American Academy of Sleep Medicine by a board certified sleep expert. Then, NoZ, SOZ, and EIZ in all patients were identified by two epileptologists. In overall, we analyzed 656 channels in NoZ (per patient: median 29, range: 3–58), 367 channels in EIZ (per patient: median: 16, range: 0–49), and 431 channels in SOZ (per patient: median: 12, range: 3–51). The number of channels in different regions for each patient is stated in Supplementary Table S1.

**FIGURE 1:**
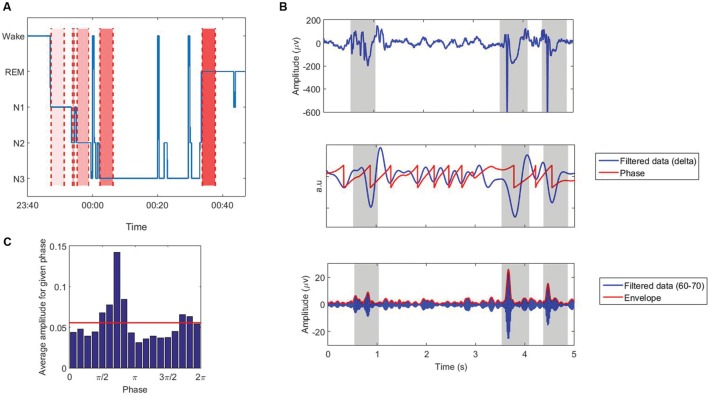
**(A)** The first sleep cycle of the hypnogram (blue line), and data selection in different sleep stages in pt. 12 as an example: N1, N2, N3, and REM: from light to dark red regions. **(B)** Extracting phase and amplitude of the signal using Hilbert transform. Shading represents intervals of sharp activities that are removed from the analysis. **(C)** Average amplitude for a given phase for the signal shown in ‘**B**’. Red line shows the uniform distribution.

During the first sleep cycle, the first 4 min of EEG signal in each stage (N1, N2, N3, and REM) was selected for processing. In a few patients, some stages were shorter than 4 min, however, we did not exclude patients with 1 or 2 stages shorter than 4 min. **Table 1** shows the number of available patients with data equal or longer than 4 min in each stage. Considering longer epochs would result in eliminating more patients especially in stage N1. Moreover, we would have to exclude at least one patient with three stages not being long enough and we would not be able to include all 25 subjects. Selected epochs had to be away from other stages for at least 15 s, meaning that we excluded the 15 s following or preceding stage changes (**Figure 1A**). We think that 4 min is long enough to avoid possible temporal variations and instability of the results, as we include more than 72 cycles of low frequency bands (0.3–30 Hz), and we also perform surrogate control analysis to confirm that the results are explained by true coupling and not by random fluctuations in the signal (see Processing).

**Table 1:** Available data in each sleep stage with at least 4 min length.

Stage	N1	N2	N3	REM
Number of patients	17	25	23	24


Wakefulness was excluded from the analysis because it was difficult to find 4 min when all patients were in the same situation (for instance they do nothing or simply watch TV). We did not find the same circumstances in the one night we had the video.

### Processing

The EEG signal was filtered in low [delta (0.3–4), theta (4–8), alpha (8–13), and beta (13–30)] and high bands [Gamma (30–80 with 10 Hz step) and ripples (80–260 with 30 Hz step)]. Filtering was performed with Brainstorm (Tadel et al., 2011). The envelope amplitude and phase of the filtered signals were then obtained by using Hilbert transform:

$$h(t)=f(t)+{if}^{\prime }(t)=A(t)e^{i\varphi \left(t\right)}$$

where *f′(t)* represents the Hilbert transform of the signal *f(t)*, and *A(t)* and φ*(t)* represent the instantaneous envelope amplitude, and instantaneous phase of the signal, respectively (**Figure 1B**). To avoid edge artifacts, the first 1000 points (long enough-confirmed by visual inspection) were removed from the analysis.

Sharp activities can generate spurious PAC (Kramer et al., 2008; Aru et al., 2015), and need to be investigated with great care. Therefore, to eliminate the effects of spikes and their following slow waves, sharp activities were detected automatically in all channels using the same method as our previous paper (Amiri et al., 2016), and 100 ms before and 400 ms after the detected sharp activities were discarded from the further analysis.

**FIGURE 2:**
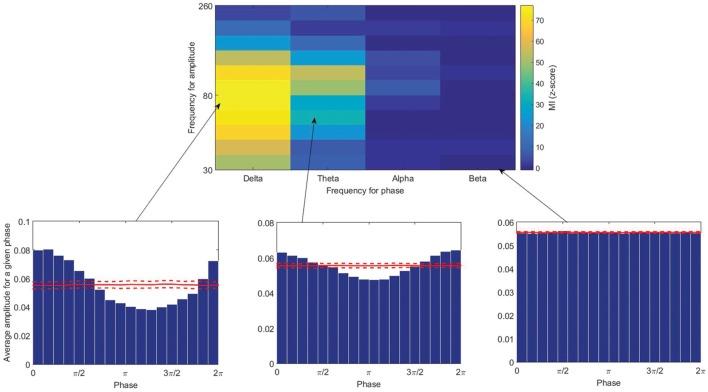
**(Top)** The modulation index calculated for different pairs of low and high frequencies. **(Below)** Average amplitude for a given phase (from left to right: high, moderate, and low couplings). Solid and broken red lines indicate the mean and the standard deviation among the 200 surrogate data.

There are many methods proposed to quantify PAC (Lachaux et al., 1999; Bruns and Eckhorn, 2004; Vanhatalo et al., 2004; Lakatos et al., 2005; Mormann et al., 2005; Canolty et al., 2006; Cohen, 2008; Osipova et al., 2008; Tort et al., 2008; Colgin et al., 2009; Kramer and Eden, 2013; Voytek et al., 2013; Dvorak and Fenton, 2014; Pittman-Polletta et al., 2014). There is no global study evaluating the performance of all of these methods, however, several studies have compared some of them (Cohen, 2008; Penny et al., 2008; Tort et al., 2010; Onslow et al., 2011; Ozkurt and Schnitzler, 2011; Kramer and Eden, 2013). Each PAC-measuring method has its own benefits and disadvantages, and there is no clear and specific gold standard; however, the modulation index (MI) proposed by Tort et al. (2008) is shown in a few studies (Tort et al., 2010; Kramer and Eden, 2013) to perform better than or equivalent to other methods in terms of tolerance to noise, independence of amplitude, and sensitivity to modulation width. In this study, PAC was calculated using this index, however, we do not think employing other methods will change the results dramatically. In this method, the Kullback–Leibler distance is computed between the amplitude distribution and a uniform distribution (**Figure 1C**). In the case of no CFC, the high-frequency amplitude distribution over the low-frequency phase bins is nearly uniform, and its distance from a uniform distribution will be small. While strong coupling leads to a clearly non-uniform distribution toward the preferred phase and the distance represents the MI (**Figure 2**). The statistical significance of the raw MI is then measured by calculating 200 surrogate MI values (Canolty et al., 2006). The phase time series is circularly shifted by a random amount (uniformly distributed between 1 and 240 s) with respect to the amplitude time series to destroy any PAC without altering the spectral power characteristics of the individual time series. The distribution of MI values derived from the surrogate procedure approaches a Gaussian distribution according to the Central Limit Theorem, so it can be used to test whether the observed raw MI is more extreme than the random surrogate data. To quantify the deviation of the raw MI from the surrogate distribution, the z-score of the MI was calculated. PAC was calculated for all low and high frequency pairs, in all channels. We did not exclude the patients who had one or two stages shorter than 4 min, and replaced the coupling values in missing stages with the corresponding values of another channel which is the closest channel in terms of Euclidean distance.

We used a mixed ANOVA to understand if there is an interaction between sleep stage and studied regions on the PAC strength. A mixed ANOVA is used when a dependent variable (in this study: PAC) is measured while all elements have undergone two or more conditions (in this study: sleep stage), and also when the elements under study have been assigned into two or more separate groups [in this study: different regions (NoZ, EIZ, SOZ)]. There are two different possibilities depending on the mixed ANOVA outputs. In case of a statistically significant interaction, we need to determine the difference between the groups at each level of each factor by using *post hoc* tests. For these tests, we implemented one way ANOVA to compare different regions at each specific sleep stage, and repeated measures ANOVA to compare different sleep stages at each specific region (NoZ, EIZ, and SOZ). However, if there is no statistically significant interaction, we need to interpret and report the main effects for both factors (in this study: sleep stage and region). We used Tukey’s method to test all possible pairwise differences of means. All statistical analysis was performed using SPSS, and all results were corrected by Tukey’s test for multiple comparisons.

It has been known for a long time that slow background activity is associated with abnormality in the EEG signal of epileptic patients (Panet-Raymond and Gotman, 1990; Gambardella et al., 1995). To test whether this increased slowing in specific channels is correlated with stronger coupling, we calculated the relative power in the delta band (
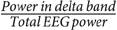
) for each channel, and assessed its correlation with the coupling strength in the same channel. We pooled all channels and subjects and calculated the correlation in each sleep stage, and then averaged the correlation of the four stages. However, as the channels of the same subject (particularly neighboring channels) are less independent than channels across subjects, we also made the alternative assumption of a repeated measure design (It is important to note that the data are not really repeated data as they are recorded from different brain regions in each patient). We recalculate the correlation under this assumption by using the patient means and finding the correlation between them (Bland and Altman, 1994).

## Results

A mixed ANOVA was conducted that examined the effect of sleep stage and region (NoZ, EIZ, and SOZ) on coupling strength. There was a statistically significant interaction between the effects of sleep stage and regions when analyzing the coupling between delta and gamma [*F*(6,3633) = 7.93, *p* < 0.01] or theta and gamma [*F*(6,3573) = 9.99, *p* < 0.01], however, there was no significant interaction between the effects of sleep stage and region in alpha and beta bands (*F*≈1). There was a significant main effect of sleep stage on the coupling strength for delta [*F*(3,3633) = 253, *p* < 0.01], theta [*F*(3,3573) = 80, *p* < 0.01], and alpha bands [*F*(3,3573) = 28, *p* < 0.01]. Furthermore, there was a significant main effect of region for all low frequency bands except for beta [delta: *F*(2,1211) = 42, theta: *F*(2,1191) = 61, and alpha: *F*(2,1191) = 33, *p* < 0.01].

The same analysis was done for the ripple band. There was a statistically significant interaction between the effects of sleep stage and different regions when analyzing the coupling between all low frequency bands, except beta, and the ripple band [for delta, theta, and alpha bands: *F*(6,3576) = 5.59, *F*(6,3549) = 10.47, and *F*(6,3648) = 3.42, *p* < 0.01 respectively and for beta band: *F*(6,3882) = 1.79, *p* > 0.01]. In the ripple band, there was also significant main effects of sleep stage [delta: *F*(3,3576) = 251, theta: *F*(3,3549) = 84, and alpha: *F*(3,3648) = 48, *p* < 0.01] and region [delta: *F*(2,1192) = 24, theta: *F*(2,1183) = 41, and alpha: *F*(2,1216) = 32, *p* < 0.01].

### PAC in Different Sleep Stages

**FIGURE 3:**
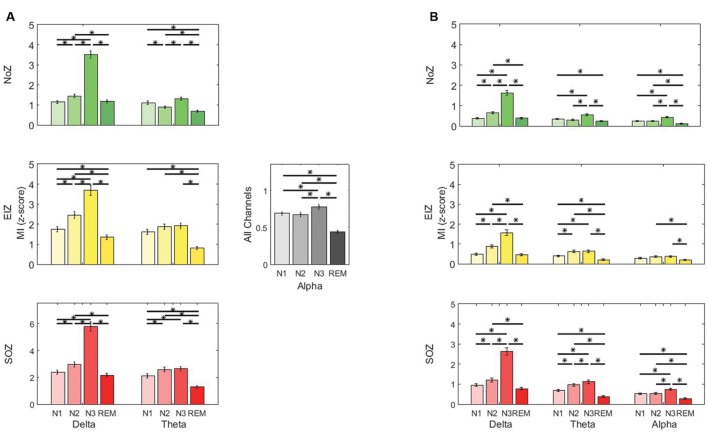
**Average phase-amplitude coupling (PAC) quantified by modulation index (MI) (**A**) gamma band and low frequencies, **(B)** ripple band and low frequencies.** Green: normal zone (NoZ), Yellow: exclusively irritative zone (EIZ), Red: seizure onset zone (SOZ), Gray: all channels. ^∗^ Shows significant difference (*p* < 0.05, corrected). The order: N1, N2, N3, REM, from light to dark color. Results are plotted as the mean ± SE.

The low frequency-gamma coupling results in different sleep stages are provided in **Figure 3A**. As there was no interaction between the effects (sleep stage and region) for the alpha band, the results in this band are shown for all channels together. Also, no significant difference was present in the beta band (*p* > 0.05), and the results are not shown. *Post hoc* simple main effects analysis showed that the coupling of low frequency and gamma band is significantly lower in REM compared to N2 and N3 in all studied regions as determined by a *post hoc* repeated measures ANOVA (*p* < 0.05, Tukey-corrected, **Figure 3A**). It is also noted that PAC in N3 is generally the highest in all regions. Particularly, in SOZ, the coupling in N3 is significantly higher compared to all other stages (except to N2 in the theta band).

The coupling of low frequency bands (delta, theta, and alpha) and ripple band was significantly higher in N3 compared to other sleep stages in SOZ and NoZ (*post hoc* repeated measure ANOVA, *p* < 0.05, Tukey-corrected). There was, however, no significant difference in the beta band (*p* > 0.05). The coupling of low frequency and ripple bands was also significantly lower in REM compared to N2 and N3 (*p* < 0.05, Tukey-corrected, **Figure 3B**) except for theta in NoZ.

### PAC in Different Regions

**FIGURE 4:**
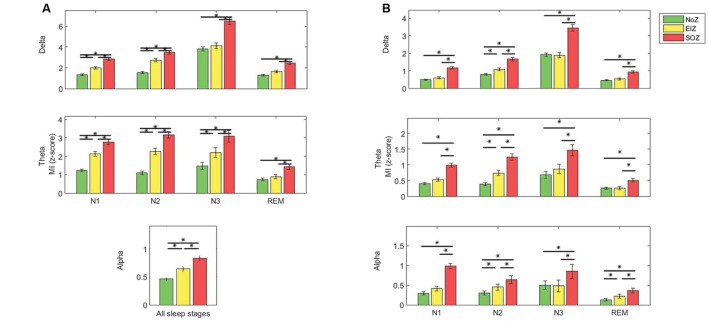
**Average PAC quantified by MI in different regions **(A)** gamma band and low frequencies, **(B)** ripple band and low frequencies (top: delta, middle: theta, below: alpha); Green: NoZ, Yellow: EIZ, Red: SOZ.** ^∗^ Shows significant difference (*p* < 0.05, corrected). Results are plotted as the mean ± SE.

*Post hoc* one-way ANOVA determined that except for the beta band, the coupling between all low and high frequency pairs was significantly stronger in SOZ compared to NoZ (*p* < 0.05, Tukey-corrected, **Figure 4**), and also in SOZ compared to EIZ (*p* < 0.05, Tukey-corrected, **Figure 4**).

The coupling strength decreased when moving from SOZ to EIZ, and to NoZ, and this result was consistent for all sleep stages and frequency pairs (except that in N3, the coupling of delta and alpha bands and ripple band was slightly higher in NoZ compared to EIZ). The coupling between low frequencies and gamma band in N1, and N2 is significantly higher in EIZ compared to NoZ (*p* < 0.05, Tukey-corrected). Note that the results of alpha-gamma coupling are cumulated for all stages due to the lack of significant interaction between effects.

The same analysis (*post hoc* one-way ANOVA followed by Tukey method) in the ripple band also revealed that the coupling between all low frequency bands and the ripple band was significantly higher in EIZ compared to NoZ in N2 (**Figure 4B**).

**FIGURE 5:**
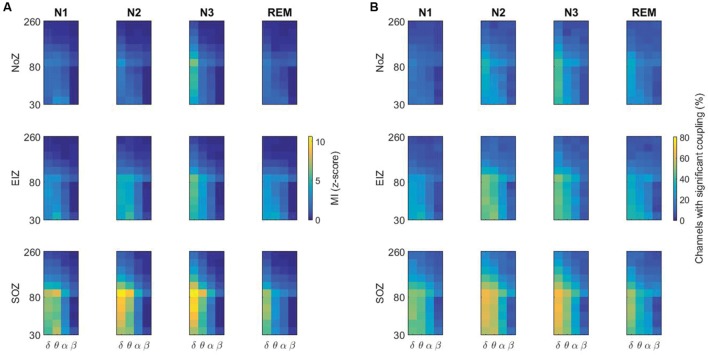
**(A)** Higher values of PAC in the SOZ compared to the NoZ, and in N3 compared to other stages. The average of PAC in all channels in different sleep stages, **(B)** Percentage of channels with significant coupling in different frequency pairs. NoZ, normal zone; EIZ, exclusively irritative zone; SOZ, seizure onset zone; MI, modulation index.

The average of PAC in NoZ, EIZ, and SOZ channels for individual patients is provided in **Supplementary Figure S1**. Overall, higher values of coupling are seen in SOZ compared to NoZ and EIZ. **Supplementary Figure S2** shows the phase-amplitude comodulogram for all channels of patient 5 as an example. **Figure 5** summarizes the results by displaying the average PAC in all SOZ, EIZ, and NoZ. As expected from the results, there are more channels in SOZ with significant values of coupling compared to EIZ and NoZ (**Figure 5B**).

### Phase Analysis

**FIGURE 6:**
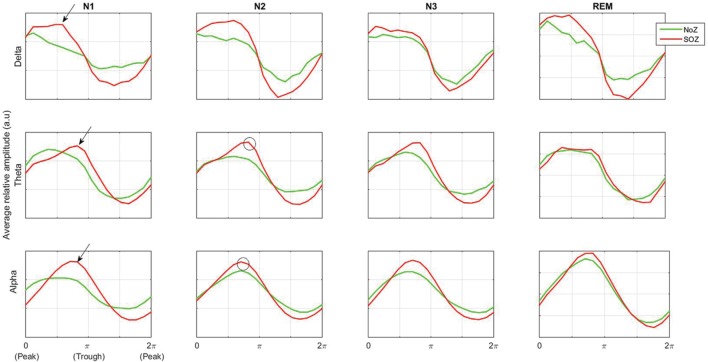
**Average ripple band amplitude as a function of low-frequency phase in normal and SOZ channels.** Phases 0 and π correspond to the low-frequency band peak and trough respectively. The difference in preferred coupling phase in theta and alpha bands compared to delta band is shown by black arrows. More high frequency activity is seen at the trough of theta and alpha rhythms (black circles). NoZ, normal zone; SOZ, seizure onset zone.

It has been shown that the density of HFOs is higher at a different phase of slow waves in epileptic channels (channels with epileptic activity, i.e., channels in SOZ or EIZ) compared to channels with normal activity, and there is a 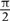 phase difference in the distribution pattern of HFOs. However, when considering the whole ripple band and not the sporadic HFOs, there is no clear difference between channels with and without epileptic activity in terms of power distribution over phase (Frauscher et al., 2015). Our results are in line with these findings. As seen in **Figure 6**, the mean power in ripple band peaks at the transition from peak to trough of delta band [transition from down to up state (Frauscher et al., 2015)] in NoZ and SOZ channels. The same general pattern is also observed in the gamma band (not shown).

Interestingly, the preferred phase of coupling in the theta and alpha band is different from that in delta band (**Figure 6**, black arrows). A similar pattern has been observed in a study on EEG activity of the hippocampus during sleep where Staresina et al. (2015) have found that the coupling of spindle (12–16 Hz) and ripple band occurs at a different phase compared to the coupling of delta and ripple bands. Furthermore, our results show that high frequency activities occur preferably at the trough of theta and alpha rhythms (**Figure 6**, black circles). In other studies, it has also been shown that high frequency activity (80–150 Hz) occurs at the trough of theta waveform in the neocortex (Canolty et al., 2006), and similarly, more high frequency activity is seen at the trough of alpha at seizure termination (Ibrahim et al., 2014).

### Background Slowing and Phase-Amplitude Coupling

**FIGURE 7:**
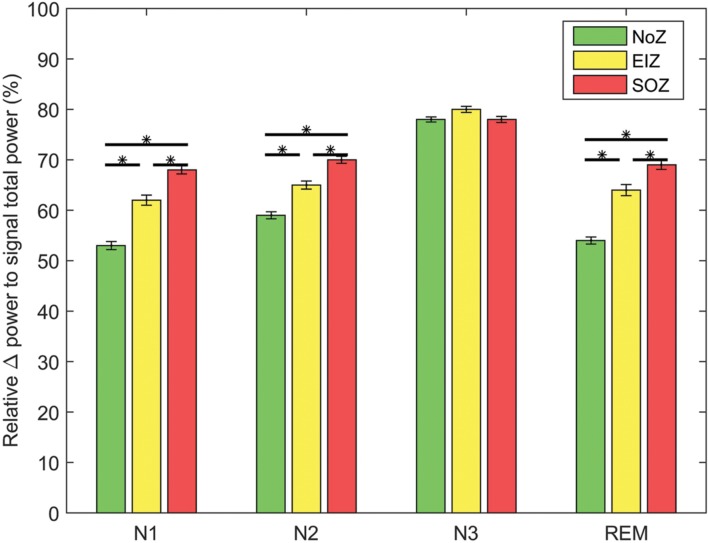
**Average of relative delta power in different regions; Green: NoZ, Yellow: EIZ, Red: SOZ.** ^∗^ Shows significant difference (*p* < 0.05, corrected). Results are plotted as the mean ± SE.

We analyzed the relative power in the delta band to examine the relation between EEG background slowing and intensity of PAC. Employing the mixed ANOVA, there was a statistically significant interaction between the effects of sleep stage and different regions [*F*(6,2676) = 28.3, *p* < 0.01]. As expected, the relative power of delta band is generally higher in SOZ compared to NoZ channels (**Figure 7**). A Pearson’s r was computed to assess the correlation between the delta-gamma coupling and the relative power of delta in each channel. Although, there was a highly significant positive correlation between the two variables in all four stages, the correlation was not strong (*r* = 0.24 ± 0.03, *p* < 0.0001). The same result was observed when studying the correlation between the delta-ripple coupling and the relative delta power (*r* = 0.22 ± 0.03, *p* < 0.0001). If we assume that the data are repeated data, the correlation between the two variables is similar, but no longer significant (for gamma band *r* = 0.25 ± 0.10, and for the ripple band *r* = 0.26 ± 0.11, *p* > 0.05).

## Discussion

It was shown that EEG dynamic variations related to the sleep-wake cycle affect the performance of seizure-prediction methods (Schelter et al., 2006) because a change in the state of vigilance is generally accompanied by a major change in the frequency spectrum of the EEG signal. Similarly, the mechanisms of gamma and theta bands modulation are shown to be state dependent (Belluscio et al., 2012). Due to the growing interest of using CFC in epilepsy studies, we studied PAC variation in different sleep stages. We observed that besides the reported variations of PAC in preictal and ictal periods (Alvarado et al., 2014; Ibrahim et al., 2014; Guirgis et al., 2015), there were significant changes in coupling when moving from one sleep stage to the other even in intervals away from seizures. We also noticed the same results in presumably normal channels. This suggests that one of the reasons underlying the variety of HFOs being modulated in different patients in previous studies might be the difference in their vigilance states (Guirgis et al., 2015). PAC studies should therefore be carried out carefully to avoid interpreting physiological PAC variation as something related to ictal or preictal episodes.

Although, we have discarded the sharp transients and therefore compared sections free of epileptic discharges (only the presumable background activity), considerable difference was seen between epileptic and normal regions. PAC may therefore represent a fundamental abnormality of the focus even outside interictal and ictal discharges. Our finding is in line with the observation of (Frauscher et al., 2016). They have also seen a significant difference in the power of high frequency bands between normal and epileptic regions even after excluding the IEDs. Many studies on analyzing the interictal EEG activity (Jacobs et al., 2008; Asano et al., 2009; Andrade-Valenca et al., 2011) have demonstrated the promising potential of interictal period in SOZ identification, here we showed that the interictal background activity could also be useful in this aspect.

The impact of sleep on epileptic activity has been addressed in many studies. For instance, sleep has been shown to modulate the HFO characteristics such as rate and duration (Staba et al., 2002; Bagshaw et al., 2009; Dümpelmann et al., 2015) and in this study we observe that it also affects the background activity.

It is interesting to note that the coupling between low and high frequency bands is higher in SOZ compared to EIZ and NoZ. The coupling is also stronger in EIZ compared to NoZ almost in all studied intervals and for all frequency pairs. This tighter coupling in SOZ has previously been reported in preictal and ictal periods in several studies (Weiss et al., 2013; Alvarado et al., 2014; Ibrahim et al., 2014; Guirgis et al., 2015). Stronger coupling in the epileptic regions may be explained by the interictal increased neuronal synchrony in these regions (Netoff and Schiff, 2002; Schevon et al., 2007; Avoli and de Curtis, 2011). In a study on microelectrodes and subdural recordings, Weiss et al. (2013) have provided some evidence that the epileptic core areas (regions fully recruited to the seizure) are associated with HFOs phase-locked to the low-frequency ictal discharges. Multi-unit activity and the amplitude of high gamma are coupled to the low-frequency phase, and therefore, high frequency activity in the core represents the excessive neuronal firing resulting from the excitatory synaptic activity in low-frequency signals (Weiss et al., 2013). PAC may therefore reflect increased action potential coherence (the hallmark of epileptic discharges at the cellular level), and may represent a measure for recruitment into epileptic discharges (Ibrahim et al., 2014). The higher coupling in SOZ and EIZ may suggest pathological action potential activities and abnormal interactions between networks (Alvarado et al., 2014). The stronger coupling of fast activities during deep sleep may also be explained by the neuronal synchrony.

The coupling measure we used in this study (MI) is independent of the signal amplitude by definition, however, it is important to note that the estimation of phase is more robust in case of high amplitude signal. Several studies have investigated interictal delta slowing, its lateralizing value and its association with surgical outcome in epileptic patients. It has been shown that slow wave background EEG activity correlates with SOZ in some patients (Gambardella et al., 1995; Tao et al., 2011) and it has a similar lateralization role to temporal spiking (Panet-Raymond and Gotman, 1990; Koutroumanidis et al., 2004; Vanrumste et al., 2005). Although, we also found higher relative power of low frequency activity in epileptic regions, there was a relatively low correlation between the coupling of low and high frequency activities and low-frequency power (using an alternate method of calculating this correlation, it was found not to be significant). With similar results, Canolty et al. (2006) have concluded that the strength of PAC (in their study: theta-high gamma) depends on low frequency power, and stronger coupling is observed in regions with higher low frequency amplitude. This is also in agreement with Tort et al. (2013) statement. Despite the statistically significant correlation between PAC and low frequency power, they are perhaps representing two different aspects of abnormality. Whether one of these measures is more localizing the epileptogenic area warrants more investigations.

In this study we did not analyze different brain regions. Nevertheless, disregarding the placement of electrodes, we still see stronger coupling in epileptic regions (**Figures 4** and **5**). Due to the large variety of patients, it is unlikely that the results are affected by a specific region. Other studies have looked at PAC in hippocampus, neocortex and thalamus without relating it to epilepsy (Canolty et al., 2006; Axmacher et al., 2010; Fitzgerald et al., 2013; Tort et al., 2013; Staresina et al., 2015). It is, however, interesting to investigate the role of PAC in epileptogenicity in these specific regions. Furthermore, considering these studies along with other studies on epilepsy as well as other diseases (for instance Alzheimer and Parkinson), physiological PAC should not be neglected besides the pathological PAC. Physiological PAC is mainly involved in brain large scale communication, learning and memory, and cognitive functions. How to distinguish these two types of PAC, and how pathology can impact physiological coupling is an open question.

We did not study the role of elevated PAC in localizing the resection area. This study was a group study, focusing on the overall variation of PAC during sleep in different brain regions. The PAC variation in each patient or each channel was not evaluated. **Supplementary Figures S1** and **S2** show some initial evidence of the capacity of PAC in determining the SOZ at the individual level. The figures demonstrate generally stronger coupling in SOZ and EIZ compared to NoZ. PAC may lead to an index for localizing epileptic brain region, and could consequently improve the surgical outcome in intractable epileptic patients. One possible direction for future research would be whether epileptogenic brain regions with high interictal PAC act differently in localizing resection area than epileptogenic areas not expressing strong PAC. Further studies are needed to determine how the resection of regions with high values of CFC correlates with seizure freedom. Whether analyzing the EEG signal in a specific sleep stage results in a more accurate localization would also be an interesting issue to be investigated in future studies.

## Author Contributions

MA designed the methods, implemented the procedure, analyzed the data and wrote the manuscript. BF collected the data, and visually scored sleep stages. JG supervised the project and revised the manuscript.

## Conflict of Interest Statement

The authors declare that the research was conducted in the absence of any commercial or financial relationships that could be construed as a potential conflict of interest.

## Supplementary Material

**FIGURE S1 | The average coupling for 25 patients in different regions.** The x-axis corresponds to low frequencies (delta, theta, alpha, beta bands) and the y-axis to the high frequencies (gamma and ripple bands). Color indicates degree of coupling and the scaling is different for each patient. Note that patients 19, 21, and 24 had no channels outside the SOZ with IEDs (blank figures). NoZ, normal zone; EIZ, exclusively irritative zone; SOZ, seizure onset zone.

**FIGURE S2 | Representative example of higher values of coupling in the epileptic channels compared to the normal channels.** PAC in all channels of patient 5 (green box: normal zone, yellow box: exclusively irritative zone, and red box: seizure onset zone), MI, modulation index.
