# Supplementary figures and images for: Phase-Amplitude Coupling Is Elevated in Deep Sleep and in the Onset Zone of Focal Epileptic Seizures
Source: Front Hum Neurosci. 2016 Aug 3;10:387. doi: 10.3389/fnhum.2016.00387 (PMC4971106; doi:10.3389/fnhum.2016.00387)

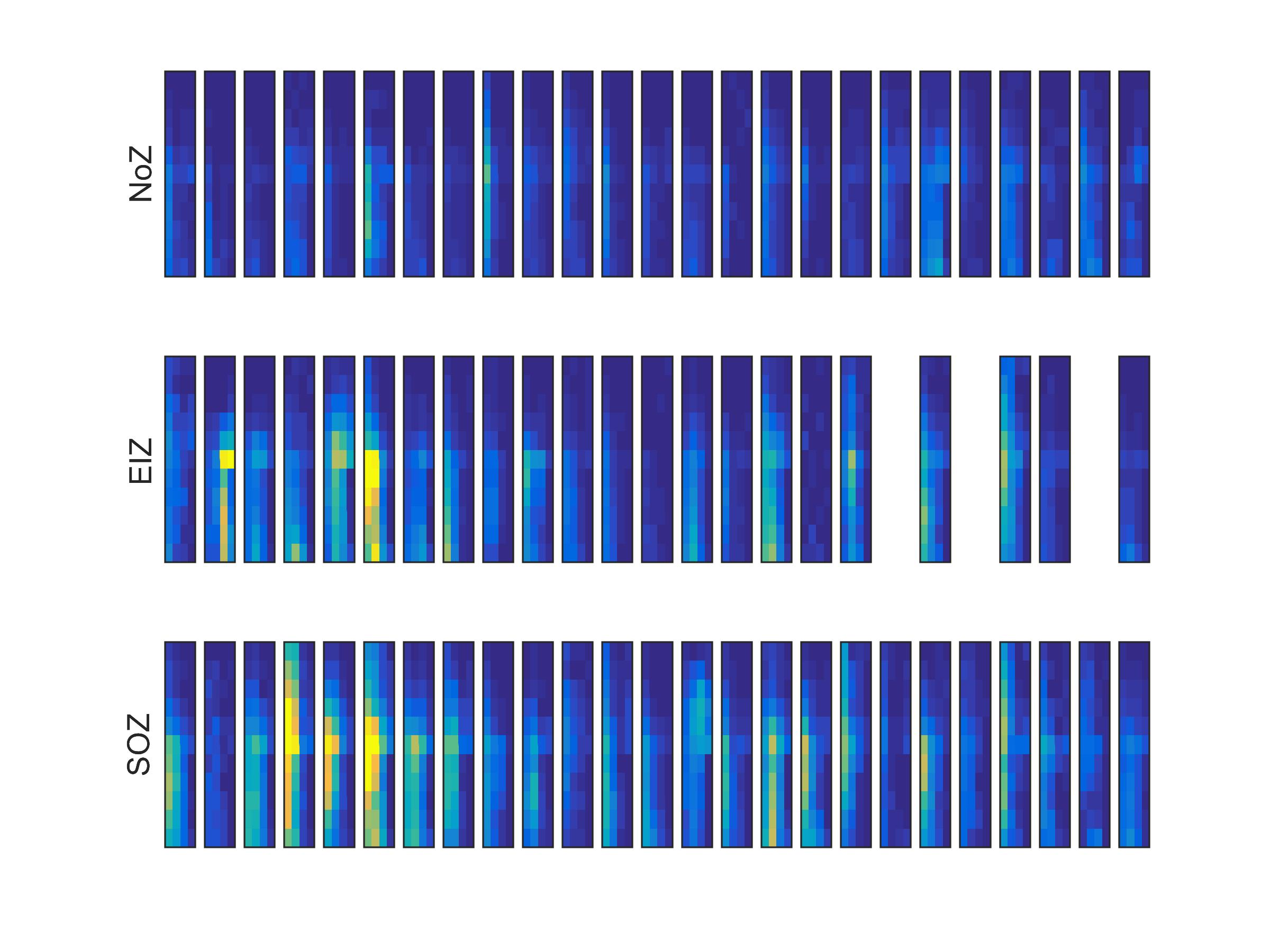

Supplement: Supplementary file 1 [file Image_1.JPEG]

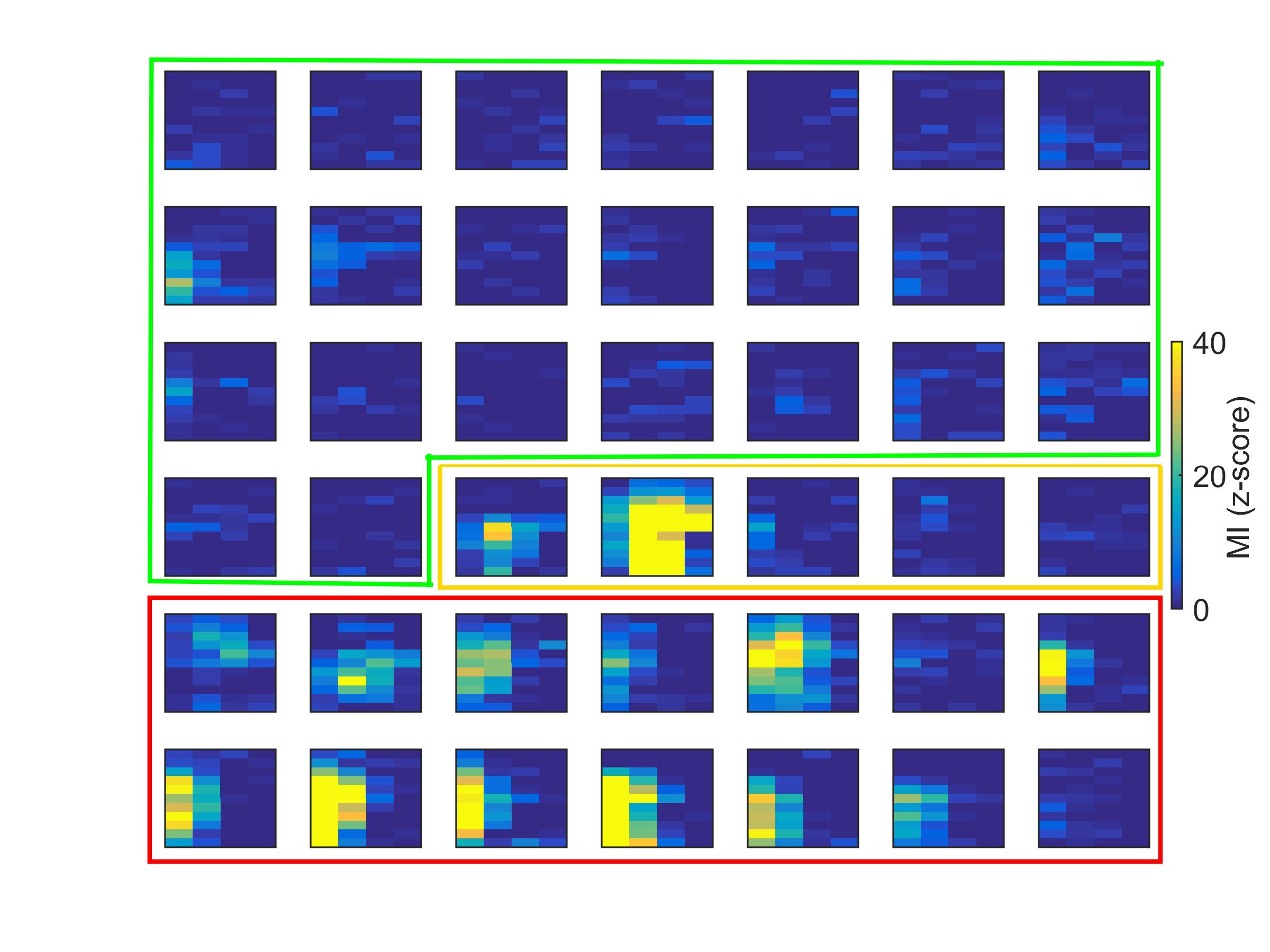

Supplement: Supplementary file 2 [file Image_2.JPEG]
