# Supplementary material for: Phase-Amplitude Coupling Is Elevated in Deep Sleep and in the Onset Zone of Focal Epileptic Seizures
Source: Front Hum Neurosci. 2016 Aug 3;10:387. doi: 10.3389/fnhum.2016.00387 (PMC4971106; doi:10.3389/fnhum.2016.00387)
Supplement: Supplementary file 3 [file Table_1.DOCX]

Supplementary Table 1. Demographic, imaging and electrophysiological data of the investigated patients

| ID | Age  /gender | MRI | Seizure onset zone | Number of electrodes | Number of channels in NoZ/ EIZ/ SOZ | Location of electrodes |
| --- | --- | --- | --- | --- | --- | --- |
| 01 | 38/m | No lesion | widespread L T and insula | 12 | 35/ 7/ 34 | L: A,H, Hp, OF, IS, CA, CM, Tp, PHp, ip,  R: H, Hp |
| 02 | 60/f | No lesion | R insula | 7 | 31/ 5/ 6 | R: OF, CA, CP, I, he, H, Hp |
| 03 | 42/f | PNH | right mesiotemporal structures (A, H), left posterior quadrant | 8 | 20/ 16/ 17 | L: A, HA, HP, CA, CP, PC, fus,  R: A |
| 04 | 48/m | No lesion | widespread left temporal plus anterior and insula | 9 | 36/ 32/ 16 | L: OF, CA, CP, IS, fus, EC, Hp, Ha, A |
| 05 | 40/m | temporo-parietal lobe gliosis | lingual gyrus L, cuneus L | 9 | 23/ 5/ 14 | L: OF, HP, CP, L, OS, P, CH, Ia, Ip |
| 06 | 38/m | polynodular heterotopia | widespread bilateral temporal L > R | 10 | 12/ 19/ 51 | L: fus, OF, CA, CP, A, Ha, Hp, R: CA, HA, HP |
| 07 | 41/m | No lesion | widespread both temporal lobes R > L | 12 | 23/ 21/ 47 | L: OF, CA, CS, A, H, Hp, R: CA, CS, Hp, H, A, OF |
| 08 | 32/f | L H atrophy | temporomesial L | 8 | 28/ 16/ 7 | L: CA, CI, CP, OI, fug, H, HP, A |
| 09 | 38/m | L H atrophy | bitemporal neocortex and left amygdala | 8 | 12/ 26/ 33 | L: H, Hp, A, Fus, R: H, A, Hp, Fus |
| 10 | 54/m | No lesion | mesio temporal and inferior isthmus R | 8 | 33/ 11/ 9 | R: A, H, Hp, cpi, Cp, PC, os, oi |
| 11 | 34/f | No lesion | temporo-occipital L | 8 | 33/ 29/ 5 | L: A, H, Hp, fus, Cp, L, R: A, H |
| 12 | 22/m | bilateral H sclerosis | mesiotemporal bothsides | 8 | 42/ 18/ 9 | L: OF, Hp, Ha, A, R: OF, A, Hp, Ha |
| 13 | 28/f | FCD in post insula and temporal transverse gyrus | post insula and transverse temporal gyrus left | 13 | 21/ 10/ 47 | L: He, Ia, Ip, PT, CP, Sms, SMi, Ha, Hp, M, S, SMA, Ca |
| 14 | 42/f | L H sclerosis | temporomesial R | 9 | 34/ 27/ 3 | L: I, Im, Hp, A, OF, R: A, H, Hp, OF, |
| 15 | 31/f | hyperintensity right frontal lobe adjecent to ventricle | Generator orbitofrontal region right (ROF, RL, RFP, RCA) | 10 | 3/ 49/ 24 | L: OF, CA, R: FP, OF, LI, CA, CM, A, H, HP |
| 16 | 33/m | encephalomalacic lesion in poterior insula area | posterior insula | 7 | 30/ 5/ 5 | L: Ia, Ip, H, P, CP, CAg, CSMg, praecentral epidurals |
| 17 | 27/f | L FCD occipital lateral | L occipital lateral | 7 | 29/ 2/ 11 | L:H, CI, OI, Os, Pc, O, Le |
| 18 | 39/m | FCD | lesion at left anterior cingulate / orbitofrontal (FCA, LOF) plus mesiotemporal (LA) | 7 | 35/ 16/ 6 | L: OF, CA, CM, H, A, R: CA, H |
| 19 | 44/m | No lesion | right central parasaggital to saggital region (SMAp + RCP) | 8 | 58/ 0/ 21 | L: SMAa, SMAp, R: SMAa, SMAp, OF, I, H, CP |
| 20 | 48/m | bilateral H atrophy, encephalomalacia frontal right | both mesiotemporal structures (H) | 8 | 33/ 9/ 14 | L: A, H, R: A, H, OF, LA, LM, LP |
| 21 | 30/f | L H sclerosis | residual hippocampus left | 6 | 23/ 0/ 3 | L:H, HP, PCu, CA, CP, I |
| 22 | 29/f | No lesion | mesiotemporal L > R | 7 | 37/ 4/ 7 | L:OF, CA, CS, A, H, R: A, H |
| 23 | 31/m | No lesion | mesiotemporal R | 6 | 9/ 20/ 6 | L:A, H, HP, R: A, H, HP |
| 24 | 45/f | No lesion | mesiotemporal L | 4 | 11/ 0/ 12 | L: H, HP, A, OF |
| 25 | 39/m | No lesion | left temporal mesial and neocortical | 6 | 5/ 20/ 24 | R:A, H, HP, L: A, H, HP, LE, RE over T1 |

Legend: m, male; f, female; R, right, L, left; F, frontal; T, temporal, H, hippocampus, A, amygdala; OF, orbitofrontal gyrus; La, encephalomalacic lesion anterior; Lm, encephalomalacic lesion mid; Lp, encephalomalacic lesion posterior; NH, nodular heterotopia; Ca, anterior cingulate gyrus; Hp, posterior part of the hippocampus; Fp, frontopolar; Cp, posterior cingulate gyrus; C, central; Cm, middle cingulate gyrus; PNH, periventricular nodular heterotopia; O, occipital; P, parietal; PC, precuneus; Ha, anterior part of the hippocampus; Fus, fusiform gyrus; Li, lingual gyrus; F2a, mid F2 lobe; SMAa, anterior part of the supplementary motor area; SMAp, posterior part of the supplementary motor area; Ia, anterior part of the insula; IS, insula superior; CM, cingulate medium; PHp, parahippocampal gyrus; CAg, angular gyrus; Os, occipital superior; Cs, Cingulate superior; I, insula; Ip, insula posterior; Oi, occipital inferior; He, Heschl gyrus; FCD, focal cortical dysplasia; PMG, polymicrogyria; Pi, inferior parietal region (supramarginal gyrus).
